# MRI texture analysis in differentiating luminal A and luminal B breast cancer molecular subtypes - a feasibility study

**DOI:** 10.1186/s12880-017-0239-z

**Published:** 2017-12-29

**Authors:** Kirsi Holli-Helenius, Annukka Salminen, Irina Rinta-Kiikka, Ilkka Koskivuo, Nina Brück, Pia Boström, Riitta Parkkola

**Affiliations:** 1 Department of Medical Physics, Medical Imaging Centre and Hospital Pharmacy, Pirkanmaa Hospital District, Post Box 2000, 33521 Tampere, Finland; 2 Department of Radiology, Tampere University Hospital, Tampere, Finland; 3 Department of Plastic and General Surgery Turku University Hospital, Turku, Finland; 4 Department of Pathology, University of Turku and Turku University Hospital, Turku, Finland; 5 Department of Radiology, University of Turku and Turku University Hospital, Turku, Finland

**Keywords:** magnetic resonance imaging (MRI), texture analysis (TA), breast cancer, invasive ductal carcinoma (IDC), volumetric analysis, prognostic factors

## Abstract

**Background:**

The aim of this study was to use texture analysis (TA) of breast magnetic resonance (MR) images to assist in differentiating estrogen receptor (ER) positive breast cancer molecular subtypes.

**Methods:**

Twenty-seven patients with histopathologically proven invasive ductal breast cancer were selected in preliminary study. Tumors were classified into molecular subtypes: luminal A (ER-positive and/or progesterone receptor (PR)-positive, human epidermal growth factor receptor type 2 (HER2) -negative, proliferation marker Ki-67 < 20 and low grade (I)) and luminal B (ER-positive and/or PR-positive, HER2-positive or HER2-negative with high Ki-67 ≥ 20 and higher grade (II or III)). Co-occurrence matrix -based texture features were extracted from each tumor on T1-weighted non fat saturated pre- and postcontrast MR images using TA software MaZda. Texture parameters and tumour volumes were correlated with tumour prognostic factors.

**Results:**

Textural differences were observed mainly in precontrast images. The two most discriminative texture parameters to differentiate luminal A and luminal B subtypes were sum entropy and sum variance (*p = 0.003)*. The AUCs were 0.828 for sum entropy (*p = 0.004*), and 0.833 for sum variance (*p = 0.003*), and 0.878 for the model combining texture features sum entropy, sum variance (*p = 0.001*). In the LOOCV, the AUC for model combining features sum entropy and sum variance was 0.876. Sum entropy and sum variance showed positive correlation with higher Ki-67 index. Luminal B types were larger in volume and moderate correlation between larger tumour volume and higher Ki-67 index was also observed (*r = 0.499, p = 0.008*).

**Conclusions:**

Texture features which measure randomness, heterogeneity or smoothness and homogeneity may either directly or indirectly reflect underlying growth patterns of breast tumours. TA and volumetric analysis may provide a way to evaluate the biologic aggressiveness of breast tumours and provide aid in decisions regarding therapeutic efficacy.

## Background

Breast cancer is known to be a heterogeneous disease that can be classified using several clinical and pathological features. Breast cancer classification may help in predicting clinical outcome, and it also has a significant role in targeting the treatment to those who are most likely to benefit. Different subtypes can be defined by using genetic array testing or approaches using immunohistochemical analyses [1]. Some of the most important factors that are related to prognosis are tumour size, histologic grade, nodal status, estrogen and progesterone receptors (ER, PR), human epidermal growth factor receptor type 2 (HER2) expressions and proliferation marker Ki-67 [2, 3]. Hormone receptor-positive breast cancers are usually classified into luminal A -like subtype and luminal B -like subtype with or without HER2 overexpression. The luminal A subtype is shown to express high levels of hormone receptor and has more favorable prognosis while, the luminal B-like subtype presents with a worse prognosis. The immunohistochemical surrogate of molecular subclasses of breast cancers proposed by the Saint Gallen Consensus Meetings [1, 4] is used to classify patients in different risk categories. In discriminating between luminal A and B subtypes, Ki-67 labeling index has been shown to be useful [5, 6]. In the era of personalized medicine, it is becoming more and more important to make a distinction between luminal A and luminal B cancers to ensure efficient treatment. The classification of molecular subtypes is done by means of genetic analysis, which is rather costly and requires specialized technical expertise. Therefore it might be beneficial to find a cost and time effective alternative means of classifying breast cancers into distinct molecular subtypes.

Breast MRI has a high sensitivity [7, 8] in diagnosing breast cancer but image interpretation still provides challenges. These challenges include misinterpretation due to technical factors [9] as well as hormonal status of the patient, difficulties in interpretation surgical [10, 11] or therapeutic interventions and [12], overlapping in the MRI appearance of some benign and malignant diseases and over-or underestimation of the lesions size [13, 14]. Breast MRI examinations generate a vast volume of image data and computer aided diagnosis systems such as texture analysis (TA) are developed to assist with lesion detection and classification. MR images contain pixel grey level variations which cannot be evaluated visually but could be detected with image analysis methods, such as TA. TA methods evaluate the spatial location and signal intensity characteristics of pixels in the images. It is a mathematical method that describes the grey level dependence between the image pixels. It offers a way to calculated mathematical values for texture features which can be used in characterizing the underlying structures of the observed tissues [15].TA has been studied as a one method to increase the specificity of breast MRI with promising results [16–20].

Breast tumors are usually heterogeneous in structure. Biopsy may often not be sufficient in assessing intratumoral heterogeneity since it does not always represent the complete phenotypic variation within a tumor. Therefore, a non-invasive method of assessing whole tumour heterogeneity might be beneficial [21]. Many studies have suggested that intra-tumoral heterogeneity can be quantified by using image texture analysis such as co-occurrence matrix (COM). Many researches have exploited texture features for distinguishing malignant from benign tumors from MR images [16–18, 22]. Though many researches had exploited COM texture features to quantify tumor heterogeneity for distinguishing malignant from benign tumors, only few very recent studies [20, 23, 24] have investigated COM feature correlation with pathological prognostic factors in invasive breast cancer. In a recent study by Sutton et al. [25] shape, texture and histogram based features were applied in differentiating breast cancer molecular subtypes with encouraging results. They divided the subtypes in ERPR+, ERPR-/HER2+ or triple negative. Since TA has proven to be potential tool in discriminating benign and malignant breast cancers, different histological types and even aid in discriminating different molecular subtypes we hypotized that it could even differentiate molecular subtypes luminal A and luminal B. Luminal A is associated with lower Ki-67 rate than subtype luminal B. Ki-67 on the other hand is associated with a higher expression of vascular endothelial growth factor in tumor cells [26] and thereby luminal B type cancers might show more heterogeneous textural appearance in MR images enabling the discrimination of the two types by the means of TA.

Larger tumors are generally associated with a poorer prognosis than smaller tumors, so tumour size can be considered as another important prognostic marker. Evaluating breast tumour size is important when determining cancer type and extent of subsequent surgical and oncological management. There are various methods to determine tumour size including palpation on physical examination and breast-imaging studies such as mammography, ultrasound, and MRI. Because of its superiority in assessing soft tissues, breast MRI is recommended to be performed also in cases where the actual tumour size cannot be estimated with other modalities [27]. In breast MR images the estimate of tumour size is usually done by measuring the largest diameter from a single slice. Volumetric analysis has also been proposed especially when studying the effectiveness of treatment [28].

Our aim was to further study the relationship between textural features and tumour volumes measured from breast MRI and molecular subtypes luminal A and B. Since one significant difference between luminal A and luminal B subtypes is a higher cell proliferation rate in luminal B types, we studied correlation between textural features, tumour volume and Ki-67. We focused our study on analyzing precontrast MR images in contrast to many previous studies which have mainly focused on analyzing texture features from postcontrast images [16–18]. Many newly diagnosed breast cancer patients undergo breast MRI and if molecular subtypes could be reliably estimated from breast MRI and provide prognostic information in addition to diagnostic imaging, the utility of preoperative breast MRI would increase.

## Methods

### Patient population

From a total of 50 consecutive adult patients referred to the Department of Plastic and General Surgery, Turku University Hospital, Turku, Finland, 27 patients were chosen in this texture analysis study. Women were eligible to participate in the study if they were 18 years of age or older and if they had received a diagnosis of unilateral invasive ductal breast cancer based on complete mammography and ultrasound workup of both breasts and ultrasound-guided core needle biopsy. Preoperatively, all patients underwent routinely breast MRI. The study protocol was approved by the Ethical Review Committee of the Hospital District of the South-West Finland. All patients gave a written consent for the study. Exclusion criteria for texture analysis study were that images were taken with the same MR scanner using the same coil and imaging sequence, no visible artifacts on MR images or lesion size less than 7 mm. Luminal A subtype (15 / 27) was defined as being ER positive, HER2 negative, and Ki-67 low (< 20% cells positive) and luminal B (12/27) subtype as being ER positive,HER2 negative or positive, and Ki-67 high (≥ 20% cells positive). From total of 50 patient only 27 met our criteria. Eight patients were excluded for not being purely ductal carcinomas, 2 were not imaged with the same MR system, 4 were not ER positive, with 6 patients the lesion size was too small and 3 had visible artifacts on MR images.

### Tumor Histology and Immunohistochemistry (IHC) analysis

Tumor type was determined on the core needle biopsy performed before chemotherapy or surgery. Four μm thick serial paraffin section were cut from tumour tissue and stained with haematoxylin and eosin. The breast cancer histology was assessed according to the World Health Organization classification [29] and tumour grading was based on the recommendations made by Elston and Ellis in 1991 [30]. Tumour grades are classified as: grade 1 is well-differentiated, grade 2 is moderately-differentiated and grade 3 is poorly-differentiated. Immunohistochemical staining of needle core biopsies for estrogen and progesterone receptors (ER, PR), Ki-67 and HER2 were performed from subsequent sections. Four different ready-to-use rabbit monoclonal antibodies were used from Ventana Medical Systems/Roche Diagnostics: Estrogen Receptor (SPI, rabbit), Progesterone Receptor (IE2, rabbit), HER2 (4B5, rabbit) and Ki-67 (30–9, rabbit) with BenchMark XT immunostainer and *ultra*VIEW Universal DAB Detection Kit (Ventana/Roche, Tucson, Arizona, USA). The percentage of nuclei with immunoreactivity to ER, PR and Ki-67 was classified as continuous data from 0 to 100%. ER-positive and PR-positive cases showed staining in at least 10% of the tumour cell nuclei. Ki-67 was defined as low if ≤ 20% Ki-67 was detected and as high if > 20% Ki-67 was detected. HER2 expression was evaluated as membrane staining of invasive tumour cells and scored from 0 to 3. Carcinomas revealing 2+ or 3+ immunostaining were retested for HER2 gene amplification with chromogenic in situ hybridization (CISH) to determine HER2 positivity. Tumour size was taken to be the diameter of the largest focus in surgical specimens. Axillary lymph node status was achieved through sentinel lymph node biopsy or axillary lymph node dissections. Table 1 presents the lesion characteristics for the 27 invasive ductal, ER positive (luminal A and B types) cases that were entered into the texture analysis.

**Table 1:** Lesion characteristics

Parameter	Patients (luminal A types)	Patients (luminal B types)
Histological type		
Invasive ductal	15	12
Histological grade		
grade 1	8	0
grade 2	7	5
grade3	0	7
Ki-67		
< 20	15	0
≥ 20	0	12
lymph node status		
negative	11	8
positive	4	4
diameter (mm)		
7 - 15 mm	9	4
≥ 15 mm	6	8

Ki-67, proliferation marker

### MRI acquisition

MR imaging was carried out on 1.5 T MRI scanner (Magnetom Avanto, Siemens Healthcare, Erlangen, Germany) using the following sequences: T2 weighted fat saturated axial-, T1 weighted fat saturated dynamic-, T1 weighted non fat saturated series in dynamic phase and diffusion imaging. Images from T1 weighted non fat saturated dynamic sequence (1.5 T: TR/TE = 8.1 ms/ 4.72 ms, acquisition time: 8.56, FOV: 320 mm, matrix 448 × 448, slice thickness 1 mm, in-plane voxel size 0.7 mm, flip angle 25 deg., number of slices 144) were used in texture analysis and volumetric measurements. The first frame was acquired before injection of paramagnetic contrast agent (Gd, 0.1 mmol/kg body, Dotarem®), followed by 5 measurements. Un-enhanced images were subtracted from the contrast-enhanced images on a pixel-by-pixel basis, creating five subtraction series.

### Image analysis

#### Texture analysis

The dynamic T1-weighted series for TA was chosen. We focused our analyses mainly on T1-weighted pre-contrast images but analyzed also the first sequence of T1-weighted postcontrast images for a comparison. Image slices were chosen on the basis of optimal representation of the largest tumour area.

**Fig. 1:**
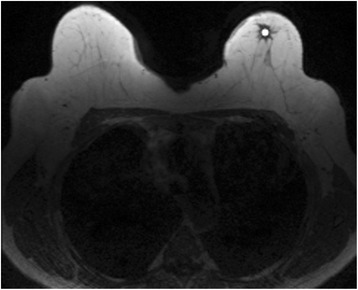
Standard size circular region of interest (ROI) loaded over T1-weighted MR image in MaZda after image normalization. The tumour is grade 3 invasive ductal carcinoma in left breast

Circular standard size regions of interest (ROI) of radius 5 pixels were positioned by hospital physicist with a special interest in developing quantitative radiology methods in clinical use. An experienced senior radiologist provided assistance for ROI settings. These regions of interest were placed into the area of the lesion where the enhancement was strongest in the first non-subtracted postcontrast image and same ROI placement was used also in pre-contrast images (Fig. 1). Standard size ROIs were used since a previous study has shown some texture features to be dependent on ROI size [31]. ROI size was chosen to ensure sufficient number of pixels for texture feature calculations and to fit all analyzed lesions to avoid partial volume effect. The grey level normalization of each standard circular ROI was performed using method which normalizes image intensities in the range [μ-3σ, μ + 3σ], where μ is the mean grey level value and σ the standard deviation, to minimize the influence of contrast variation and brightness [32].

For TA we used a specialized program MaZda package version 4.6 (The Technical University of Lodz, Institute of Electronics). MaZda allows computation of texture parameters based on image histogram, co-occurrence matrix, run-length matrix, image gradients, autoregressive model and wavelet analysis [33]. In this study we used the 2D co-occurrence (COM) based parameters since, according to our experience, they have shown potential in breast MRI TA studies. The co-occurrence matrix is a second order histogram of an image and it relates into groups of pixels or pixel pairs. The basic element of grey level co-occurrence matrix is the count of the pixel pairs which have a certain grey level value in given direction and pixel distance. Rather than using gray level co-occurrence matrix directly in texture analysis, the co-occurrence matrices can be converted into scalar measures of texture, which can be used to measure the textures of images and regions In total, 11 COM-based (angular second moment, contrast, correlation, sum of squares, inverse difference moment, sum average, sum variance, sum entropy, entropy, difference variance and difference entropy) were calculated with the distance of one pixel and in four directions (0°, 45°, 90°, and 135°). The four directional components of each parameter were averaged into one parameter in order to enhance the robustness of the method. Angular second moment is a measure image uniformity. This feature obtains a high value, when a grey level distribution in the image is either constant or periodic. Thus a higher value for this feature indicates that the image is homogenous. Also feature inverse difference moment measures image homogeneity. When inverse difference reaches high values, image can be considered to be smooth. Contrast measures the local variations in the image. The lowest contrast value can be obtained when the pixels have the same or very similar grey level values. Correlation measures the grey level linear dependencies in the image. It measures how well correlated one pixel is with its neighbor over the whole image. If an image has a large areas of similar intensities, correlation will be high. Sum of squares is the variance of the co-occurrence matrix and the values are somewhat similar to the values of histogram variance. Sum average gives the average of sums of two pixel values in the image of interest. The pixel pairs which are used in calculation are the ones used in forming the co-occurrence matrix, thus the sum average is not dependent on the direction or the on the pixel distance used in calculations. Entropy- based features indicate the complexity and randomness within the region of interest. Difference variance and difference entropy are based on differences calculated between two pixel values [33].

#### Tumour volume

Volumetric analysis was done with image postprocessing software ImageJ, which is a public domain, Java-based image processing program developed at the National Institutes of Health [34]. In all T1-weighted subtracted images tumour mass was manually outlined on the computer monitor. The area of tumour in each slice was multiplied by the slice profile, and total tumour volume was automatically calculated by summation of the adjacent volumes.

### Statistical analysis

Statistical analysis was performed using commercially available software (SPSS, v. 22.0; Chicago, IL). Because of skewed distribution of the data, the independent Mann–Whitney *U*-test was used to determine whether the texture features calculated from pre- or postcontrast images were significantly differing between luminal A and luminal B cancers. To further evaluate how different and able to separate both groups TA parameters were, empirical ROC curves were generated for the parameters which presented significant differences between luminal A and luminal B types. The area under the curve (AUC) which measures how well a parameter can distinguish between two diagnostic groups (AUC close to 1 indicate a very informative test) was calculated. The texture features which proved to be statistically different between luminal A and B types and were not strongly correlated with each other were used as an entry in a binary logistic regression. Additional ROC curve was computed to assess the accuracy of a predictive model. ROC curves were used to identify optimal cutoff values in distinguishing luminal types. In order to assess the generalization error, leave-one-out cross validation (LOOCV) was performed. Spearman correlation was used to study how texture features correlated with Ki-67 or grade or volume. The values of r, 0–0.19 is regarded as very weak, 0.2–0.39 as weak, 0.40–0.59 as moderate, 0.6–0.79 as strong and 0.8–1 as very strong correlation. The resulting *p*-values less than 0.05 are considered to be statistically significant.

## Results

### Texture features and volumes between luminal A and luminal B

Luminal A and B types presented differing textural appearance. Most of the texture features calculated from precontrast images were significantly different. Postcontrast images however did not yield so promising results (Table 2). Luminal types were also significantly differing in volume *(p = 0.041)*, luminal B types tended to be slightly larger.

**Table 2:** Textural differences between luminal types A and B. Calculated from pre- and postcontrast images

		Precontrast		postcontrast	
	Texture feature	*p*-value	Higher value in group A/B	*p*-value	Higher value in group A/B
Texture difference between luminal A and luminal B	Angular second moment	**0.016**	luminal A	0.212	luminal A
	Contrast	**0.025**	luminal A	0.322	luminal A
	Correlation	**0.007**	luminal B	0.212	luminal B
	Sum of squares	0.126	luminal B	0.527	luminal B
	Inverse different moment	0.829	luminal A	0.118	luminal B
	Sum average	0.093	luminal B	0.595	luminal B
	Sum variance	**0.003**	luminal B	0.145	luminal B
	Sum entropy	**0.003**	luminal B	0.145	luminal B
	Entropy	**0.021**	luminal B	0.231	luminal B
	Difference variance	**0.007**	luminal A	0.595	luminal A
	Difference entropy	**0.021**	luminal B	0.118	luminal B

Significant *p*-values (*p* < 0.05) are given in bold face

**Fig. 2:**
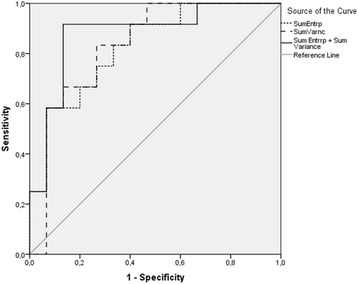
ROC curves of texture analysis parameters in distinguishing between luminal A and luminal B types. Sum entropy (*dotline*), Sum variance (*dash line*), and predictive model combining sum entropy and sum variance (*solid line*) are shown. Diagonal line represents AUC of 0.50. The ROC curves represent the complete data set, please refer to text for LOOCV results. AUC values are given in Table 3.

The AUC obtained from the ROC curves were calculated for all significantly differing texture features and obtained values were near 0.7 in all analyses. The texture features sum entropy and sum variance presented the highest AUC (*Sum entropy, 0.828; Sum variance, 0.833*). These two features were used in a binary logistic regression as they did not have strong correlation with each other *(r = 0.447, p = 0.022).* Additional ROC curve was computed to assess the accuracy of a predictive model with these two most discriminative texture parameters. The results of ROC curve analysis representing the complete data set for sum entropy, sum variance and the model combining these parameters are shown in Fig. 2 and Table 3. The combination of sum entropy and sum variance resulted in an AUC equal to 0.878 to correctly characterize luminal B type (*p* = 0.001). For combination of these two parameters, a cutoff value of 0.497 resulted in a sensitivity of 91.7% and specificity of 86.7% to separate both groups**.** The diagnostic values barely decreased in the cross validation: The LOOCV resulted in 91.3% sensitivity and 86% specificity. Adding tumour volume in the prediction model did not yield any better outcome. As for comparison empirical ROC curves were generated also TA features calculated from postcontrast images, even though they did not statistically differ between luminal A and B types. The AUC obtained from the ROC curves were all under 0.5 (*Sum entropy, 0.327; Sum variance, 0.327*).

**Table 3:** ROC curve AUC values of texture features sum entropy, sum variance and their combination (precontrast images)

texture feature	AUC, Mean ± SD	*p*-value	AUC, Mean ± SD (LOOCV)
Sum entropy	0.828 ± 0.079	**0.004**	0.827 ± 0.065
Sum variance	0.833 ± 0.081	**0.003**	0.832 ± 0.076
Sum entropy + Sum variance	0.878 ± 0.72	**0.001**	0.876 ± 0.077

Significant *p*-values (*p* < 0.05) are given in bold face

*AUC* area under the curve, *LOOCV* leave-one-out cross validation

### Correlations between texture features and prognostic features

There were 8 G1 lesions, 12 G2 lesions and 7 G3 lesions. A higher grade tumours showed tendency to be a slightly larger in volume (*p = 0.05*). No notable correlations were observed in TA parameters calculated from pre-or postcontrast images between different grade tumours. Only one feature, difference variance, showed moderate negative correlation with tumour grade (*r* = −0,469, *p* = 0.03) other correlation coefficients were all under 0.4 and *p* values over 0.05.

Moderate correlation between tumour volume and Ki-67 index was observed (*r = 0.499, p = 0.008*), indicating that larger tumours had higher Ki-67 index. Most TA parameters calculated from precontrast images correlated with Ki-67 index. Sum entropy and sum variance both correlated with Ki-67 index (*sum entropy: r = 0.607, p = 0.001; sum variance: r = 0.5, p = 0.008*). Sum entropy also seemed to correlate positively with tumour volume (*r = 0.637, p < 0.001*). Entropy-based TA features from postcontrast images seemed also correlated with Ki-67 index. However correlations were only moderate (sum entropy: *r* = 0.447, *p* = 0.40; entropy: *r* = 0.391, *p* = 0.44; diff entropy: *r* = 0.5, *p* = 0.07).

## Discussion

We showed in this study that texture analysis could discriminate luminal A and luminal B types of ductal carcinomas. In this study we combined TA and tumour volumetric analysis in studying if we can differentiate the two ER positive cancers; luminal A and luminal B types. Malignant tumours possess more heterogeneous tissue architecture [35] and therefore might reflect a wider and more heterogeneous range of pixel values in MR images. Our hypothesis was that more aggressive cancers might also present greater degree of textural heterogeneity in the MR images.

Several calculated texture features from T1-weighted precontrast images were significantly different (*p* < 0.05) between luminal A and B types. Significantly differing features in our study; angular second moment, contrast, correlation, sum variance, sum entropy, difference variance and difference entropy are all one way or another, measures of heterogeneous textural appearance. According to our study luminal B type cancer have in fact more heterogeneous appearance in MR images than luminal A types. Especially entropy-based features or features representing heterogeneous and random textural patterns have also shown potential in discriminating benign and malignant breast tumours in breast MR images in previous studies [17–19]. Most previous studies have applied texture analysis in postcontrast MR images [16–20, 22]. By calculating texture features from postcontrast images one may captivate mostly the texture pattern caused by the spreading and distribution of the contrast media. One of our aims was specifically to study the textural appearances and differences between luminal A and B types in precontrast MR images, to reveal the underlying tissue architecture. In our study T1-weighted postcontrast images did not show significant textural differences between luminal A and B types. One possible reason for this might be ROI size, which was relatively small. The signal from the contrast media might have partially masked the real underlying texture pattern.

Luminal A and luminal B types showed also difference in tumour volume (*p = 0.041*). Luminal B types were larger in volume than luminal A types. Larger tumours tended to correlate positively with entropy based features (*entropy r = 0.682, p < 0.001 and sum entropy r = 0.637, p < 0.001*) and negatively with angular second moment (*r = − 0.65, p < 0.001*). Since entropy is a measure of randomness and heterogeneity of the studied region and correspondingly angular second moment represents homogeneity of the given region, it appeared that larger tumours are more heterogeneous and complex on their texture appearance. The Ki-67 index is correlated with a high mitotic count and recurrent disease [36]. In our study, a high Ki-67 index was related to higher values in entropy based features (*sum entropy: r = 0.607, p = 0.001*).

Since histologic grade is a characteristic feature when evaluating tumour aggressiveness, we studied also the effect of histological grade on tumour volume and texture features. A slightly greater tumour volume was observed in higher grade tumours. However, since only one texture feature correlated with tumour grade (*difference variance, r = −.419, p = 0.3*), we may think that tumour grade is not a dominant factor on revealed textural differences.

MRI texture analysis has been used in discriminating benign and malignant lesions [16] classifying the underlying breast cancer subtypes [20, 37] and also evaluating treatment response [38, 39]. There seems to be a growing interest in studying the potential of TA in shedding light to histopathologic features [20, 24, 40] and providing further information on the biologic aggressiveness of breast tumours. Tumour heterogeneity and its relationship with pathology have been studied [16, 20]. Ahmed et al. recently observed textural differences between triple negative breast cancers and other types in contrast-enhanced MRI [41] and in their work Bhooshan et al. showed that co-occurrence textural features measured in contrast-enhanced MRI could distinguish in situ ductal carcinoma and invasive ductal carcinoma, and also invasive ductal carcinoma with positive lymph node. The authors found that lesion heterogeneity, which can be presented using texture features, was an indicator of malignancy [42]. In a more recent study by Bhooshan et al. [43] applied texture analysis of DCE-MRI breast images with other computerized methods such as kinetic features in an attempt to distinguish between invasive ductal carcinoma lesions of Grade 1, Grade 2, and Grade 3 with very encouraging results.

According to our study the proliferation rate and, in some extend the size of the tumour, correlated with the texture features. Our previous study [31] has shown that many, especially entropy-based features, are dependent of ROI size. Therefore in this study we used standard size and shape ROIs to eliminate the possible effect of ROI size to our texture measurements. Though it was not in fact an immense discovery that larger and higher grade tumours possess more heterogeneous textural appearance, it is an interesting finding that luminal A and B types proved to be different in volume and in texture. The study did not have a very large patient group due to the fact that we wanted to select homogeneous material. Nevertheless we were still able to identify statistically significant differences in textural features between luminal A and luminal B subtype. We acknowledge that the limitation of this study is the small sample size and texture features which were approaching significance also in T1-weighted postcontrast images may become statistically significant with a larger sample size. Features calculated from postcontrast images which did achieve smallest *p*-values were features which represent heterogeneous patterns and were in fact the same ones which did statistically differ in precontrast images (Table 2). It is necessary to also note that this study contained high number of comparisons and since *p*-values are not corrected for multiple comparisons there might be some false positive findings due to random variability. A future study of interest would be to further assess the performance of texture features especially with MR images without contrast media, with larger data set and proper classification analysis with an appropriate training set. Also the use of other MR sequences would be one of our future interests. Combining information from both T2-weighted and T1-weighted MR images for distinguishing different kind of breast lesions might be advantageous. Also another limitation of this preliminary study is that due to the small sample size we did not perform a separate validation set for the logistic regression model and the performance of the model remains somewhat unclear. A further evaluation of the model needs to be explored in future work on a larger data set and also combining magnetic resonance (MR) imaging kinetic and morphologic features to the analysis.

## Conclusions

In conclusion, our results indicate that textural features of pre-contrast T1w images may be used for sub- typing of breast cancer. Texture features which measure randomness, complex or smoothness and homogeneity are the ones which seem to differentiate luminal A and luminal B types. These MR image texture features may either directly or indirectly reflect underlying growth patterns and, therefore, may prove useful in decisions regarding therapeutic efficacy and in the monitoring and follow-up of breast cancers during and after treatment. Differentiating between luminal A and luminal B breast cancers is nowadays important for treatment planning. The immunohistochemical classification of breast cancer as a clinical tool is supported because it can be used at a reasonable cost. However, biopsy represents just a small area of the tumour volume. Thus, a non-invasive analysis like TA offers a method to assess the whole tumour volume and could be of great value in assisting in treatment decision.

## Abbreviations

CAD: Computer aided diagnosis
COM: Co-occurrence matrix
ER: Estrogen receptors
HER2: Human epidermal growth factor receptor type 2
MR: Magnetic resonance
MRI: Magnetic resonance imaging
Ki-67: Proliferation marker Ki-67
PR: Progesterone receptors
ROI: Regions of interest
TA: Texture analysis
